# Low vitamin D level was associated with metabolic syndrome and high leptin level in subjects with nonalcoholic fatty liver disease: a community-based study

**DOI:** 10.1186/s12876-019-1040-y

**Published:** 2019-07-16

**Authors:** Li-Wei Chen, Cheng-Hung Chien, Sheng-Fong Kuo, Chia-Ying Yu, Chih-Lang Lin, Rong-Nan Chien

**Affiliations:** 1 Department of Gastroenterology and Hepatology, Chang-Gung Memorial Hospital and University at Keelung, 12F, No 222, Mai-Jin Road, Keelung, Taiwan 20401; 2 Community Medicine Research Center, Chang-Gung Memorial Hospital and University at Keelung, 12F, No 222, Mai-Jin Road, Keelung, Taiwan 20401; 3 Metabolism and Endocrinology, Chang-Gung Memorial Hospital and University at Keelung, Keelung, Taiwan

**Keywords:** Vitamin D, Non-alcoholic fatty liver disease, Metabolic syndrome, Insulin resistance, Fatty liver, Ultrasonography, Adiponectin, Leptin

## Abstract

**Background:**

This study aimed to evaluate the association between serum vitamin D levels and nonalcoholic fatty liver disease (NAFLD) parameters, such as metabolic syndrome (MS), inflammatory cytokines (tumor necrosis factor, high sensitive C-reactive protein) and adipokines (adiponectin, leptin).

**Methods:**

From August 2013 to August 2016, a community-based study was performed in the north-eastern region of Taiwan. All subjects received a demographic survey, blood testing and abdominal ultrasonography (US). The vitamin D level was evaluated by quartile divide or used the classification of deficiency (< 20 ng/ml), insufficiency (20–30 ng/ml) and sufficiency (> 30 ng/ml).

**Results:**

Subjects were divided into NAFLD group and normal control (subjects number = 564 in each group) following abdominal US study and matching age and gender. The mean age was 57.1 years in NAFLD group and 57.5 in control group. Subjects in NAFLD group had a lower mean vitamin D than those in the control group (28.5 ± 9.5 ng/ml vs. 29.9 ± 10.2 ng/ml, *P* = 0.018). Subjects with serum vitamin D deficiency or insufficiency had higher odds for MS than those with sufficient vitamin D levels [deficiency vs. sufficiency, adjusted odds ratio (aOR) =1.860 (95% CI = 1.234–2.804), *P* = 0.003; insufficiency vs. sufficiency, aOR = 1.669 (95% CI = 1.237–2.251), *P* = 0.001]. Similarly, subjects in the lowest quartile of vitamin D had higher odds for MS than those in the highest quartile of vitamin D (aOR = 2.792, 95% CI = 1.719–4.538, *P* < 0.001). Vitamin D level was positively correlated with age and male, but negatively correlated with serum leptin level.

**Conclusion:**

Subjects with low vitamin D level had higher odds for MS, but higher levels of leptin, compared to those with high vitamin D levels.

## Introduction

Nonalcoholic fatty liver disease (NAFLD) is defined as fat accumulation in the liver greater than 5% by weight in the absence of excessive alcohol consumption [1, 2]. NAFLD is reported to be associated with other systemic diseases, such as cardiovascular diseases, insulin resistance (IR), obesity, dyslipidemia and metabolic syndrome (MS) [1–4]. The prevalence of NAFLD is increasing and has been estimated to be between 10 and 30% worldwide [5, 6]. Vitamin D deficiency is also a worldwide condition and is present in approximately 30–60% of the general adult population [7, 8]. Vitamin D levels have been reported to be inversely related with fasting glucose concentrations [9, 10], adiposity [11, 12], lipid [13], and blood pressure [14–16]. Fasting blood sugar, adiposity (central obesity), lipid and blood pressure are the component of MS criteria. Previous studies have revealed that vitamin D deficiency can significantly increase the risks of IR and MS [8, 17, 18]. Vitamin D may influence hepatocytes and non-parenchymal hepatic cells (hepatic stellate cells, Kupffer cells) in NAFLD via metabolic, anti-inflammatory and anti-fibrotic effects [19, 20]. Several inflammatory cytokines, such as interleukin (IL)-6, tumor necrosis factor alpha (TNF-α) and IL-1β, and adipokines, such as adiponectin and leptin, may participate in these reactions [19, 20]. Because NAFLD and vitamin D deficiency engender similar risks for cardiovascular disease, insulin resistance and MS in epidemiologic studies, there have been many reports debating a potential association between NAFLD and vitamin D deficiency [19–23]. However, most studies have lacked information on serum cytokines or adipokines and had small sample sizes. The hypothesis in this study was that vitamin D influences MS and NAFLD via inflammatory cytokines [such as TNF-α and highly sensitive C reactive protein (HS-CRP)] and adipokines (such as adiponectin and leptin). The primary aim in this study was to analyze the potential association between vitamin D level, MS and NAFLD from community-based cohort data. The secondary aim was to reflect that the association between serum vitamin D levels and levels of adiponectin, leptin, HS-CRP and TNF-α.

## Materials and methods

From August 2013 to August 2016, a community-based survey for MS was performed in the Wanli, Ruifang and Anle districts in the north-eastern region of Taiwan. The Institutional Review Board of the Chang-Gung Memorial Hospital approved this research (IRB No: 103-3886C). All participants agreed to the study conditions and provided written informed consent before enrollment in this study. Informed written consent was obtained from all subjects.

Because the majority of MS develops in people with middle age [24], the inclusion criteria were: subjects aged > 30 years who underwent abdominal ultrasonography (US) and completion of blood testing. Exclusion criteria were:

1. Current pregnancy.
2. Alcoholic liver disease (Men who consumed > 30 g alcohol /day and women who consumed > 20 g alcohol/day).
3. Secondary causes of steatosis (e.g., corticosteroid use, gastric bypass surgery).
4. Seropositivity for hepatitis B surface antigen (HBs Ag) or anti-hepatitis C virus antibody (anti HCV).
5. Underlying metabolic liver disease, such as Wilson disease, genetic hemochromatosis, and autoimmune diseases.
6. People who took vitamin D supplementation within 3 months prior to enrollment.

All subjects participated in a demographic survey, physical examination and blood testing. Blood tests included complete blood cell counts, liver and renal biochemistry tests, fasting glucose and insulin levels, vitamin D, adiponectin, leptin, TNF-α and HS-CRP levels and lipid profiles. The demographic survey assessed the past history of systemic diseases, such as hypertension, diabetes mellitus, hyperlipidemia, hematologic disorders, autoimmune diseases, medication history, and family history. The physical examination evaluated heart rates, blood pressures, body weights, body heights, and waist girths (circumferences). Body mass index (BMI) (kg/m^2^) was calculated as weight (kg) divided by squared height (m). Subjects were asked to fast overnight before giving blood samples. Blood samples were analyzed within 4 h after collection to determine complete blood cell counts, biochemical parameters, and antibody titers. The serum samples were stored in tubes at − 80 °C following centrifugation (3000 rpm at 4 °C for 30 min). The assays for adiponectin and leptin were performed using stored serum samples.

### Serum vitamin D

Serum concentrations of vitamin D (25-hydroxyvitamin D [25(OH)D]) were measured by radioimmunoassay (Vitamin D total, Roche Diagnostics, Mannheim, German) according to the manufacturer’s instructions. An electrochemiluminescence binding assay was performed using Elecsys and Cobas immunoassay analyzers, with measurement ranges of 3.00 to 70.0 ng/mL and 7.50 to 175 nmol/L, respectively. Current study was conducted in Keelung city (latitude: 25∘N), where sunshine was adequate all the year. Hence, serum vitamin D was checked all the year without avoid the winter time. Vitamin D status was defined based on the traditional classifications of “deficient” (< 20 ng/mL, level 1), “insufficient” (20–30 ng/mL, level 2), and “sufficient” (> 30 ng/mL, level 3) [25]. The other evaluation was quartiles divided the data of vitamin D values into quartile 1–4 (quartile 1, Q1:< 22.41 ng/ml, quartile 2, Q2: 22.4–28.41 ng/ml, quartile 3, Q3: 28.41–35.38, quartile 4, Q4:> 35.38 ng/ml). Using the subjects with serum vitamin D level among sufficiency (> 30 ng/ml) or Q4 (> 35.38 ng/ml) as the reference, a logistical regression analysis was applied to elucidate the associations between vitamin D level and metabolic syndrome or hepatic steatosis.

### Adiponectin and leptin levels

Levels of adiponectin and leptin were evaluated using commercial kits (Human Total Adiponectin/Acrp30, BioVendor Research and Diagnostic system, Minneapolis, MN; Human Leptin ELISA, Clinical Range, BioVendor Laboratory Medicine, Karasek, Czech Republic) according to the manufacturers’ instructions. The method of analysis was a quantitative sandwich enzyme immunoassay.

### Tumor necrosis factor alpha (TNF-α)

The TNF-α assay used a quantitative sandwich enzyme immunoassay technique and was performed according to the manufacturer’s instructions (Immunite 1000 LKNF1, Siemens Medical Solutions Diagnostics, Llanberis, UK).

### Homeostasis model assessment of insulin resistance (HOMA-IR)

Insulin resistance was assessed using the homeostatic model assessment (HOMA-IR) score after excluding participants with DM [26]. The HOMA-IR score was calculated with the following formula:

$$ \mathrm{Fasting}\ \mathrm{plasma}\ \mathrm{insulin}\ \left(\mathrm{mU}/\mathrm{L}\right)\ \mathrm{x}\ \mathrm{fasting}\ \mathrm{plasma}\ \mathrm{glucose}\ \left(\mathrm{mmol}/\mathrm{L}\right)/22.5 $$

A higher HOMA-IR score corresponds to lower insulin sensitivity [27]. Because there was no fixed value of HOMA-IR for insulin resistance analysis, we also applied the value of HOMA-IR 2 and 3 for further analysis.

### Metabolic syndrome (MS)

A race-specific waist girth threshold based on the NCEP ATP III criteria was utilized to prevent distortions in MS prevalence [28, 29]. The cut-off values for normal waist circumference in Asian men and women were set to 90 cm (35.5 in.) and 80 cm (31.5 in.), respectively.

### NAFLD fibrosis score

The NAFLD fibrosis score was calculated according to the following formula: − 1.675 + 0.037 × age (yr) + 0.094 × BMI (kg/m2) + 1.13 × impair fasting glucose (IFG)/diabetes (yes = 1, no = 0) + 0.99 × AST/ALT ratio – 0.013 × platelet (× 10^9^/L) – 0.66 × albumin (g/dL) [30]. A score lower than the low cutoff score (− 1.455) may exclude advanced fibrosis (F0–2) (negative predictive value of 93%), and a score greater than the high cutoff score (0.676) may predict the presence of advanced fibrosis (F3–4) (positive predictive value of 90%). A score within the range − 1.455 to 0.676 is recognized as indeterminate.

### Estimated glomerular filtration rate (eGFR)

The Modification of Diet in Renal Disease (MDRD) Study equation was applied to evaluate the kidney function. The MDRD Study equation is given below [31]:

$$ \mathrm{GFR}\ \left(\mathrm{mL}/\min /1.73\;{\mathrm{m}}^2\right)=175\times {\left(\mathrm{Serum}\ \mathrm{Cr}\right)}^{-1.154}\times {\left(\mathrm{Age}\right)}^{-0.203}\times \left(0.742,\mathrm{if}\ \mathrm{female}\right)\times \left(1.212,\mathrm{if}\ \mathrm{African}\ \mathrm{American}\right) $$

### Fatty liver evaluation

Liver ultrasonography (US) (Toshiba, Japan) was performed to assess the extent of fatty liver disease by two operators who were blinded to laboratory values. The degree of fatty liver disease was graded as normal (absent), mild, moderate or severe on the basis of the intense reflection level of echogenicity (brightness) arising from the hepatic parenchyma with liver-kidney contrast, far attenuation sign by echo penetration into the deep portion of the liver and obscuring changes of the vessel wall and gallbladder wall [32–34].

### Data analysis and statistics

For continuous variables, values are expressed as the means ± standard deviations (SD). Categorical data were analyzed with the Chi-squared test or Fisher’s exact test, as appropriate. All statistical tests were 2-tailed. A *P*-value < 0.05 was considered to indicate a statistically significant difference. Phi coefficient was applied for binary data, such as with or without fatty liver conditions; Spearman’s coefficient rho was applied for a nonparametric measure of rank correlation, such as vitamin D levels (normal, insufficiency, deficiency or quartile); Pearson’s correlation was applied for continuous data, such as HS-CRP, TNF-α, adiponectin, and leptin levels. Multivariate logistic regression analysis was performed after adjusting for the potential confounders of age, sex and body mass index. Statistical analyses were performed using SPSS software for Windows (Version 16.0, SPSS Inc., Chicago, IL).

## Results

**Fig. 1:**
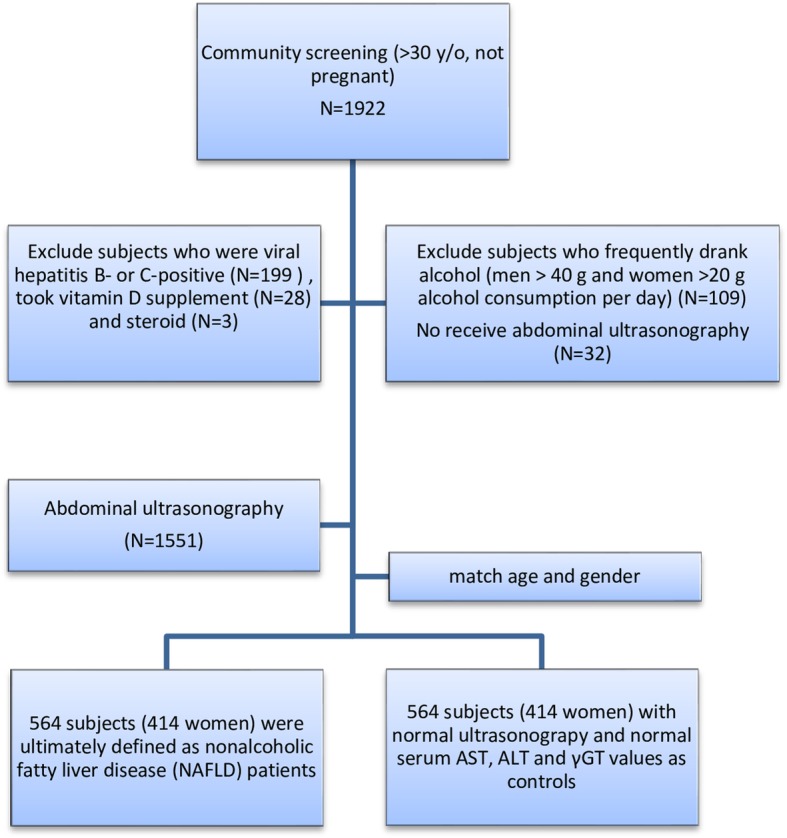
Study flow diagram

Initially, 1922 participants were enrolled. Participants who were viral hepatitis B- or C-positive (199 subjects), frequently drank alcohol (men > 30 g and women > 20 g alcohol consumption per day, 109 subjects), took vitamin D supplement (28 subjects) or steroid treatment (3 subjects), and declined to take abdominal US study (32 subjects) were excluded. According to the abdominal US findings, fatty liver change was found in 835 subjects (544 women) and the other 716 (537 women) subjects with normal US study. After matching age and gender, finally 564 subjects with fatty liver but no viral or alcoholic liver diseases were included in NAFLD group. The other 564 subjects with normal US finding, and normal serum AST, ALT and gamma-glutamyl transpeptidase (GGT) values were included as controls in this study (Fig. 1). The mean age was 57.1 ± 11.9 years in the NAFLD group and 57.5 ± 11.9 years in the control group (*P* = 0.618). The demographic and characteristic data are listed in Table 1. The prevalence of MS was higher in the NAFLD group than in the control group (202/564, 35.8% vs. 87/564, 15.4%, *P* < 0.001). Subjects in the NAFLD group also had higher mean values of weight, waist circumferences, systolic/diastolic blood pressure, triglycerides, sugar, AST, ALT, and leptin, but lower mean values of HDL and adiponectin, than the mean values of subjects in the control group. The percentage of subjects exhibiting IR according to the HOMA-IR index was greater in the NAFLD group than in the control group, no matter used the cutoff point of HOMA-IR at 2, 2.5 or 3. The mean value of vitamin D was lower in the subjects with NAFLD (28.5 ± 9.8 ng/ml) than the mean value in control group (29.9 ± 10.2 ng/ml) (*P* = 0.018).

**Table 1:** Demographic and clinical characteristics of total participants

	NAFLD	Control	*P* value
Number	564	564	
Mean age (years)	57.1 ± 11.9	57.5 ± 11.9	0.618
Gender			1.000
Male	150 (26.6)	150 (26.6)	
Female	414 (73.4)	414 (73.4)	
BMI (kg/m^2^)	26.0 ± 3.5	23.3 ± 3.4	< 0.001
DM	117 (20.7)	49 (8.7)	< 0.001
Waist Circumference (cm)	82.9 ± 9.3	76.3 ± 8.8	< 0.001
FBG (mg/dL)^a^	104.9 ± 24.4	97.9 ± 20.7	< 0.001
TG (mg/dL)^a^	156.1 ± 446.5	95.4 ± 58.0	0.001
HDL (mg/dL)^a^	54.0 ± 13.3	60.3 ± 15.1	< 0.001
Metabolic syndrome	202 (35.8)	87 (15.4)	< 0.001
HOMA-IR value^a^	2.5 ± 2.4	1.6 ± 2.9	< 0.001
HS-CRP (mg/L)^a^	2.1 ± 3.9	1.6 ± 4.5	0.030
Adiponectin (ng/mL)^a^	6.7 ± 4.3	10.2 ± 5.7	< 0.001
Leptin (ng/mL)^a^	12.8 ± 7.8	9.1 ± 6.2	< 0.001
TNF-α (ng/mL)^a^	6.0 ± 2.3	5.9 ± 2.2	0.749
WBC,×10^3^ cells/uL^a^	6.1 ± 1.7	5.5 ± 2.0	< 0.001
Vitamin D (ng/ml)^a^	28.5 ± 9.5	29.9 ± 10.2	0.018
AST (U/L)^a^	25.7 ± 17.8	21.7 ± 4.4	< 0.001
ALT (U/L)^a^	27.5 ± 27.6	18.4 ± 5.7	< 0.001
Alkp (mg/dL)^a^	68.8 ± 26.4	64.4 ± 17.5	0.001
BilT (mg/dL)^a^	0.8 ± 0.3	0.9 ± 0.3	0.265
γGT (U/L)^a^	24.7 ± 20.6	17.5 ± 10.0	< 0.001

*BMI* body mass index, *DM* diabetic mellitus, *FBG* fasting blood glucose, *HOMA-IR* homeostasis model assessment of insulin resistance, *HDL* high density lipid, *TCHOL* total cholesterol, *LDL* low density lip, *HS-CRP* high-sensitivity C reactive protein, *TNF-α* Tumor necrosis factor alpha, *WBC* white blood cell, *i-PTH* intact parathyroid hormone, *Alkp* alkaline phosphatase, *BilT* total bilirubin, *γGT* gamma glutamyl transpeptidase,

^a^data presented as the mean ± standard deviation

Table 2 shows the correlation between vitamin D level and other factors, such as age, gender, BMI, adiponectin, leptin, TNF-αand HS-CRP. It reveals that vitamin D level was positively correlated with age and male, but negatively correlated with leptin.

**Table 2:** Correlation between Vitamin D level (by classification or quartile) and other factors

Variable	Classification^a^	Quartile^b^
Age	0.332*	0.335*
Male	0.233*	0.274*
BMI	0.032	0.017
TNF-α	0.028	0.018
HS-CRP	−0.007	0.006
Adiponectin	0.053	0.032
Leptin	−0.124*	−0.175*

^a^Vitamin D classification: normal (> 30 ng/ml), insufficiency (20–30 ng/ml), deficiency (< 20 ng/ml)

^b^Vitamin D (quartile): quartile1 (< 22.41 ng/ml), quartile2 (22.41–28.41 ng/ml), quartile3 (28.41–35.38 ng/ml), quartile4 (> 35.38 ng/ml)

*P < 0.05, by Spearman’s rank correlation coefficient

Table 3 shows the logistical regression analysis for the association between vitamin D level and MS, after adjusting for the confounding factors of age, gender, BMI and NAFLD status. Subjects with deficient or insufficient vitamin D levels (< 20 or 20–30 ng/ml) had a higher odds for MS when compared with those with sufficient vitamin D levels (> 30 ng/ml) [deficiency vs. sufficiency, adjusted OR = 1.770 (95% CI = 1.145–2.736), *P* = 0.010; insufficiency vs. sufficiency, adjusted OR=, 1.673 (95% CI = 1.220–2.295), *P* = 0.001). When using the quartile method of vitamin D, the result of associations between vitamin D level and MS was similar with the method by deficiency, insufficiency and sufficiency classification. Subjects with vitamin D value located in Q1 (< 22.41 ng/ml) had a higher risk of MS (OR = 2.473, 95% CI = 1.580–3.871, *P* < 0.001) than those with vitamin D value located in Q4 (> 35.38 ng/ml). Similarly, subjects with vitamin D value located in the Q2 (22.41–28.41 ng/ml) or Q3 (28.41–35.38 ng/ml) also had a higher risk of MS than those with vitamin D value located in Q4. When a subgroup analysis was performed for subjects with NAFLD, subjects with low vitamin D level still had a trend of increasing risk of MS than those with high vitamin D level. Likewise, subjects with vitamin D value located in Q1 had a higher risk of MS (OR = 2.358, 95% CI = 1.386–4.013, *P* < 0.001) than those with vitamin D value located in Q4.

**Table 3:** Association between Vitamin D level and metabolic syndrome

	Adjusted OR (95% CI)					
All^a^	Insufficiency vs. normal		*P*-value	Deficiency vs. normal		*P*-value
Metabolic syndrome	1.673 (1.220–2.295)		0.001	1.770 (1.145–2.736)		0.010
NAFLD^b^						
Metabolic syndrome	1.633 (1.125–2.369)		0.010	1.607 (0.962–2.685)		0.070
All^a^	Q1 vs. Q4	*P*-value	Q2 vs. Q4	*P*-value	Q3 vs. Q4	*P*-value
Metabolic syndrome	2.473 (1.580–3.871)	< 0.001	1.896 (1.243–2.893)	0.003	1.881 (1.243–2.846)	< 0.001
NAFLD^b^						
Metabolic syndrome	2.358 (1.386–4.013)	0.002	1.858 (1.113–3.103)	0.018	1.972 (1.202–3.235)	0.007

*OR* odds ratio, CI = 95% confidence interval,

Vitamin D levels: normal (> 30 ng/ml), insufficiency (20–30 ng/ml), deficiency (< 20 ng/ml).

Vitamin D quartile: Q1 (< 22.41 ng/ml), Q2 (22.41–28.41 ng/ml), Q3 (28.41–35.38 ng/ml), Q4 (> 35.38 ng/ml).

^a^adjusted for confounding factors: age, gender, BMI and NAFLD status

^b^adjusted for confounding factors: age, gender and BMI

In view of the association between vitamin D level and NAFLD status, vitamin D insufficiency or deficiency did not increase the risk of having NAFLD when compared with sufficient vitamin D levels [insufficiency vs. sufficiency: adjusted OR = 1.096 (95% CI = 0.839–1.432, *P* = 0.501); deficiency vs. sufficiency: adjusted OR = 1.238 (95% CI = 0.804–1.775, *P* = 0.245)].

Table 4 shows the distribution between vitamin D levels and the severity of fatty liver disease by abdominal US. There was no significant difference in the percentage of vitamin D level among the mild, moderate and severe fatty liver by abdominal US (*P* > 0.05 by Chi-squared test).

**Table 4:** The distribution of Vitamin D levels by the severity of fatty liver disease measured by abdominal ultrasonography

Fatty liver	Mild	Moderate	Severe	*P-* value
Vitamin D				0.199
Normal	118 (20.9)	100 (17.7)	14 (2.5)	
Insufficiency	119 (21.1)	89 (15.8)	24 (4.3)	
Deficiency	60 (10.6)	33 (5.9)	7 (1.2)	
Vitamin D				0.058
Q1	85 (15.1)	55 (9.8)	10 (1.8)	
Q2	77 (13.7)	49 (8.7)	15 (2.7)	
Q3	70 (12.4)	63 (11.2)	17 (3.0)	
Q4	65 (11.5)	55 (9.8)	3 (0.5)	

Vitamin D classification: normal (> 30 ng/ml), insufficiency (20–30 ng/ml), deficiency (< 20 ng/ml)

Vitamin D quartile: Q1 (< 22.41 ng/ml),Q2 (22.41–28.41 ng/ml), Q3 (28.41–35.38 ng/ml), Q4 (> 35.38 ng/ml)

When a NAFLD fibrosis score was applied for liver fibrosis evaluation, most subjects with NAFLD were not found to have an advanced fibrosis status. Regarding the NAFLD fibrosis score, 77.0% of subjects had a NAFLD fibrosis score < − 1.455, indicating a low degree of fibrosis (F0–2). Only 6 subjects with a NAFLD fibrosis score > 0.675 (high degree of fibrosis, F3–4) were detected in this study. The mean value of vitamin D in these 6 subjects was 39.0 ± 10.9 ng/ml (range: 20.1 to 52.4 ng/ml). Because of the small number of subjects with advanced liver fibrosis in this study, we could not draw any firm conclusions regarding an association between vitamin D level and fibrosis in subjects with NAFLD.

## Discussion

Recent studies have revealed that vitamin D has a broad effect on immune modulation, cell differentiation, proliferation and inflammation regulation. Possible mechanisms to explain the association between vitamin D level and NAFLD include vitamin D-mediated improvement in insulin secretion and insulin resistance, decreased adipose tissue inflammation, and decreased hepatic inflammation and fibrosis via the regulation of the vitamin D receptor and several cytokines, such as interleukin 6, TNF-α or adiponectin [19, 35, 36]. The hypothesis of the current study was that vitamin D influences the status of MS via inflammatory cytokines (TNF-α and HS-CRP) or adipokines (adiponectin and leptin). In bivariate correlation study, vitamin D level was not associated with TNF-α, HS-CRP or adiponectin levels. But vitamin D level was negatively correlated with leptin. Previous studies reported vitamin D-mediated inhibition of leptin [37]. Another study reported increased leptin level was linked to decreased HDL-C level [38]. In current study, subjects with vitamin D deficiency and NAFLD had increased leptin value but lower HDL-C level. It could partially explain the association between low vitamin D level and MS in subjects with NAFLD. A further study of the potential associations among vitamin D, leptin, MS and NAFLD is warranted [19–21, 38, 39].

Manson’s et al. reported an over-estimation of vitamin D deficiency and unnecessary vitamin D supplement by applying the cut point of < 20 ng/mL as a deficiency status for general people [40]. They suggested using estimated average requirement (EAR) for the general population. Hence, the definition of vitamin D deficiency might use a different cut point of serum vitamin D value for people in the different areas [40, 41]. Because the real EAR value is unavailable in our area, we used both traditional classification (deficiency, insufficiency or adequate) and quartile divide (Q1–4) methods to evaluate vitamin D status. This analysis revealed a consistent result of MS risk by these two methods.

Participants in NAFLD group have a higher mean BMI and waist but lower vitamin D than those in control group. A decreased release of stored vitamin D from adipose tissue into the circulation and decreased cutaneous synthesized vitamin D3 into circulation induce lower serum vitamin D in obese subjects [14, 42]. Rhee et al. have reported that serum vitamin D levels were lower in the group with NAFLD compared to the control group [43]. Targher et al. also reported a lower serum vitamin D level in those with biopsy diagnosed NAFLD than normal controls (20.4 ± 8.8 vs. 29.8 ± 6 ng/mL, *P* < 0.001) [44]. Therefore, several published studies have found evidence of a lower serum vitamin D level in subjects with NAFLD compared to those without NAFLD [45]. However, there have been several studies reporting conflicting results concerning vitamin D level and NAFLD status. One Chinese study by Li L et al. [46] and another Korean study by Hong et al. [47] reported no significant differences in vitamin D levels between groups with or without NAFLD. The reasons for these conflicting results regarding a potential association between vitamin D level and NAFLD status may include the different methods and criteria for NAFLD and vitamin D deficiency diagnosis, selection bias in cross-sectional studies and no adjustment for confounding factors, such as BMI. Moreover, polymorphisms of the vitamin D receptor gene and the interaction between downstream products and vitamin D concentration may be associated with the progression and severity of liver diseases [48, 49]. Hence, vitamin D may influence the development or the progression of NAFLD only in certain individuals with specific genotypes.

The current study has similar limitations as previous cross-sectional studies. First, a selection bias may exist in this community-based screening study. Most subjects participating in this study were women and of older age. Second, the definition of hepatic steatosis severity and NAFLD fibrosis scores were made by abdominal US and calculated formulae, respectively, without histological confirmation. The current study was based on a community cohort, and liver biopsy was not feasible or practical. During advanced fibrosis or cirrhosis, steatosis and necroinflammatory reactions may disappear in patients with NASH, and this status is known as “burn-out NASH” [50]. Patients with NAFLD but advanced liver fibrosis may have no sign of fatty liver in the abdominal US because of burning out NASH. In our study, advanced fibrosis (score > 0.674) by NAFLD fibrosis score could be detected in both NAFLD group and normal group. We could not identify whether the subjects with advanced fibrosis in normal group are burn-out NASH or not. Moreover, the prevalence of advanced fibrosis was low in our NAFLD subjects, which was similar with other Asia studies [51, 52]. Because of low number in patients with NAFLD and advanced liver fibrosis, we could not draw any conclusions regarding an association between vitamin D levels and fibrosis in subjects with NAFLD. Third, the test of serum vitamin D was checked all the year without avoid the winter time. Current study was conducted in Keelung City (latitude: 25∘N), where sunshine was adequate all the year. Keelung is unlike some areas in high latitude (above the 37∘N latitude), where skin vitamin D production decreases because of reduced ultraviolet B (UVB) exposure during winter (November to February) [53]. In conclusion, subjects with low vitamin D levels have an increased odds of metabolic syndrome compared to those with sufficient or high vitamin D levels. The distribution of fatty liver severity (as measured by abdominal US) was similar in subjects with NAFLD among the different vitamin D levels.

## Data Availability

The datasets generated and analyzed during the current study are not publicly available because they are community-based data and are not approved for public demonstration by our institutional review board. However, these data are available from the corresponding author on reasonable request.

## Abbreviations

aOR: Adjusted odds ratio
BMI: Body mass index
EAR: Estimated average requirement
GGT: Gamma-glutamyl transpeptidase
HOMA-IR: Homeostasis model assessment of insulin resistance
HS-CRP: High-sensitivity C-reactive protein
IR: Insulin resistance
MS: Metabolic syndrome
NAFLD: Nonalcoholic fatty liver disease
TNF-α: Tumor necrosis factor alpha
US: Ultrasonography
